# Physical literacy-based intervention for older adults: a cluster randomized controlled trial study protocol

**DOI:** 10.3389/fspor.2024.1392270

**Published:** 2024-07-17

**Authors:** Raymond Kim Wai Sum, Yijian Yang, Siu Ming Choi, Michael J. Duncan, Minghui Li

**Affiliations:** ^1^ Department of Sports Science and Physical Education, Faculty of Education, The Chinese University of Hong Kong, Hong Kong, Hong Kong SAR, China; ^2^ Faculty of Education, University of Macau, Taipa, Macao, Macao SAR, China; ^3^ Centre for Sport, Exercise and Life Sciences, Faculty of Health and Life Sciences, Coventry University, Coventry, United Kingdom; ^4^ Faculty of Physical Education, Ningbo University, Ningbo, China

**Keywords:** physical literacy, older adults, randomized clinical trial, study protocol, Hong Kong

## Abstract

The ageing population creates concerns and challenges worldwide. The large number of older adults (aged over 65) in Hong Kong continues to rise as people live longer. This may result in heavy burdens on public services and problems such as a shortage of medical resources. The purpose of this study is to implement a physical literacy-based intervention among older adults in Hong Kong in order to achieve the goal of health promotion. A two-arm cluster randomized controlled trial will be employed in this proposed study. Ten daycare centers for the older adults in Hong Kong will be invited to participate in this study. The intervention group will receive functional fitness training and mastering physical literacy class twice a week with buddy peer support, and they will be asked to keep a reflective writing journal on a daily basis for 12 weeks in total. Participants will be evaluated at baseline (week 0), post-intervention (week 12), and at 6-week follow-up (week 18). This will consist of objective and self-reported measures covering elements within physical literacy (i.e., physical competence, motivation and confidence, knowledge and understanding) and also physical activity levels on an individual basis. The study intends to introduce a conceptual framework of physical literacy for the older adults through an intervention that allows older people to develop daily behaviour habits, which should promote active ageing for the older adults and greater self-esteem in later life. After this study, participants may share their positive experiences, and encourage their peers in the community to become physically literate in the future. In the long run, due to the feasibility and sustainability of these potential programs, this proposed study has the potential to connect seniors through social engagement and contribute to healthy living. Clinical trial approval from the National Library of Medicine (Reference number: NCT06137859).

## Introduction

The global phenomenon of an ageing population presents a myriad of concerns and challenges, demanding attention and proactive measures worldwide. However, despite this pressing issue, scant attention has been paid to the development of physical literacy among older adults, both globally and specifically within the unique context of Hong Kong. Older adults continue to age, and the global population aged 65 years or above will increase significantly from 10% in 2022 to 16% by the end of 2050 (1). Particularly by 2050, the percentage of people aged 65 years or above is projected to account for 40.6% of the total population in Hong Kong which makes Hong Kong the highest share of people aged 65 or above in the world (1). The burden on public services and the strain on medical resources could be alleviated by promoting initiatives and programs aimed at fostering healthier, more independent, and active lifestyles among older adults in Hong Kong.

### Embracing the concept of physical literacy among older adults

Central to this endeavor is the concept of physical literacy, which encompasses “the motivation, confidence, physical competence, knowledge, and understanding necessary for individuals to value and take responsibility for engaging in physical activities throughout their lives” (p. 8) (2). While initially rooted in educational discourse as far back as 1938, the concept of physical literacy has garnered renewed attention and acceptance, transcending disciplinary boundaries and gaining global recognition (3). Unlike traditional notions of physical activity, strength, or fitness, the concept of physical literacy acknowledges the holistic nature of human embodiment, encompassing the physical, cognitive, and affective domains. A physical literacy journey refers to an individual's ongoing commitment to the continuous physical activity participation, and it underscores the lifelong journey of acquiring and maintaining a disposition towards physical activity, regardless of age or innate abilities (4). Therefore, the development of physical literacy is the culmination of a lifelong endeavor and beneficial for all the older adults (5).

### The role of physical literacy in healthy ageing

For older adults, cultivating physical literacy is especially crucial as it enables them to effectively navigate the physical challenges associated with ageing, injury, and chronic disease, thereby maintaining independence and functional autonomy for an extended period (6). Older adults who are physically literate can adapt to challenges associated with injury, chronic disease, and aging, thereby engaging in continuous participation in physical activity compared to those who engage in less physical activity. Through the lens of physical literacy, successful ageing entails adeptly adjusting and modifying activities, optimizing motivation and enjoyment of movement, and sustaining higher levels of functioning across all dimensions (7). Thus, initiating an active lifestyle early on not only preserves independence but also contributes to overall well-being across the lifespan, aligning with the goal of promoting healthy ageing (2).

### Significance in the development of physical literacy, health promotion and gerontology

This study seeks to introduce a conceptual framework of physical literacy through a Physical Literacy-Based Intervention (PLBI), aimed at fostering good exercise habits and daily behavioral routines among older adults. By tailoring interventions to individual physical literacy journeys and lifestyles, this approach offers a meaningful pathway towards active ageing, empowering older adults to realize their potential for physical literacy in later life. Additionally, evidence suggests that group exercise promotes better physical and mental health among older adults, highlighting the potential for community-wide benefits from such interventions (8). Peer support among older adults will also encourage each other in the community to engage in physical activity in the long run. Meanwhile, as the concept of physical literacy has been vaunted as a key component in the establishment of lifelong adherence to physical activity, it is important to implement a physical literacy-based intervention for older adults. McLennan and Thompson (9) also espouse that physical literacy is the foundation of quality physical education. The development of physical literacy is the most influential variable with respect to physical health improvement. The proposed research, therefore, aims to implement a PLBI including functional fitness training and mastering physical literacy class, buddy peer support, and reflective writing for the development of motivation, confidence, physical competence, knowledge and understanding under the concept of physical literacy to achieve the goal of health promotion. This will add an effective comprehensive assessment and the charting of physical literacy for older adults. In the short term, this study will lead to a convenient and powerful program and assessment tool that can be used by older adults centers and older adults fitness trainers and assessors. In the long term, this research will benefit the general public, in particular, communities of older people by encouraging their participation in physical activity. When framing the proposed model to chart physical literacy for older adults, this program and measurement system may be adopted in older adults and ageing population in the future studies.

### Objectives of study

The study will aim to (1) develop and implement a PLBI including functional fitness training, mastering physical literacy class, buddy peers support, and reflective writing for older adults in Hong Kong; (2) explore physical literacy (physical competence, daily behavior, knowledge and understanding, and motivation and confidence) among the older adults in Hong Kong; (3) examine the effectiveness of a PLBI in terms of changes in physical literacy development among older adults in Hong Kong; and (4) add information to the literature of physical literacy, gerontology, and public health in the Asian context of Hong Kong.

### Hypothesis

We hypothesize that after the intervention and follow-up:

(1) The increase in physical competence of participants in the intervention group will be greater than their counterparts in the control group.
(2) The increase in physical activity engagement levels of participants in the intervention group will be greater than their counterparts in the control group.
(3) The increase in self-reported physical activity levels, knowledge and understanding, physical literacy, and motivation and confidence of participants in the intervention group will be greater than their counterparts in the control group.

## Methods and materials

The Physical Literacy Interventions Reporting Template (PLIRT) (presented in Table 1) has been established to support researchers and practitioners in planning, reporting, and interpreting physical literacy-based intervention (10). It was also designed to meet only the specificities of the physical literacy concept. In this regard, this study protocol will be guided by the PLIRT for the reporting of study results. The proposed study has been granted clinical trial approval from the National Library of Medicine *(Reference number: NCT06137859)* and ethical approval from the Survey and Behavioral Research Ethics Committee of the Chinese University of Hong Kong (*Reference number: SBRE-21-0353*) to collect and distribute samples and data from the participants.

**Table 1:** The PLIRT checklist.

Item Nr.	Description	Location in the paper and/or comment
Title		
1	Highlight the role of PL in the title	
Background and definition		
2	Describe the relevance of PL for the target population/group/individual	
3	Explain your conceptualization of PL and refer to a holistic definition of PL	
4	Formulate PL-related goals/aims of your study	
Assessment		
5a	*If quantitative:* Choose a multidimensional assessment strategy of PL and provide information about psychometric properties	
5b	*If qualitative:* Develop a qualitative method that closely aligns with PL theory and the different domains	
Design and content		
6	Ensure that your interventional approach is in line with PL-compatible philosophical assumptions	
7	Mention the intervention provider(s), describe his/her/their expertise specific to PL, and any specific training given	
8	Report in detail intervention content related to all PL domains	
9	Explain whether and how you realized the integrative arrangement of content/techniques	
10	Consider general guidelines for intervention reporting	
Evaluation		
11	Describe how the PL intervention was accepted and/or whether it was implemented as intended (modifications, fidelity, compliance, adherence)	
12a	*If quantitative:* Report transparently how the different PL domains (and, if initially intended, other relevant outcomes such as health) were affected by the intervention	
12b	*If qualitative:* Characterize the strengths, weaknesses, and challenges of your PL intervention; the different PL domains may help you structure the analysis and results	
Discussion and conclusion		
13	Discuss the limitations of your PL intervention, especially whether (if yes, where and why) you had to deviate meaningfully from your planned conceptualization	
14	Break down your experiences with the PL intervention and derive sound recommendations for future studies	

PLIRT, physical literacy interventions reporting template; PL, physical literacy.

### Study design

**Figure 1:**
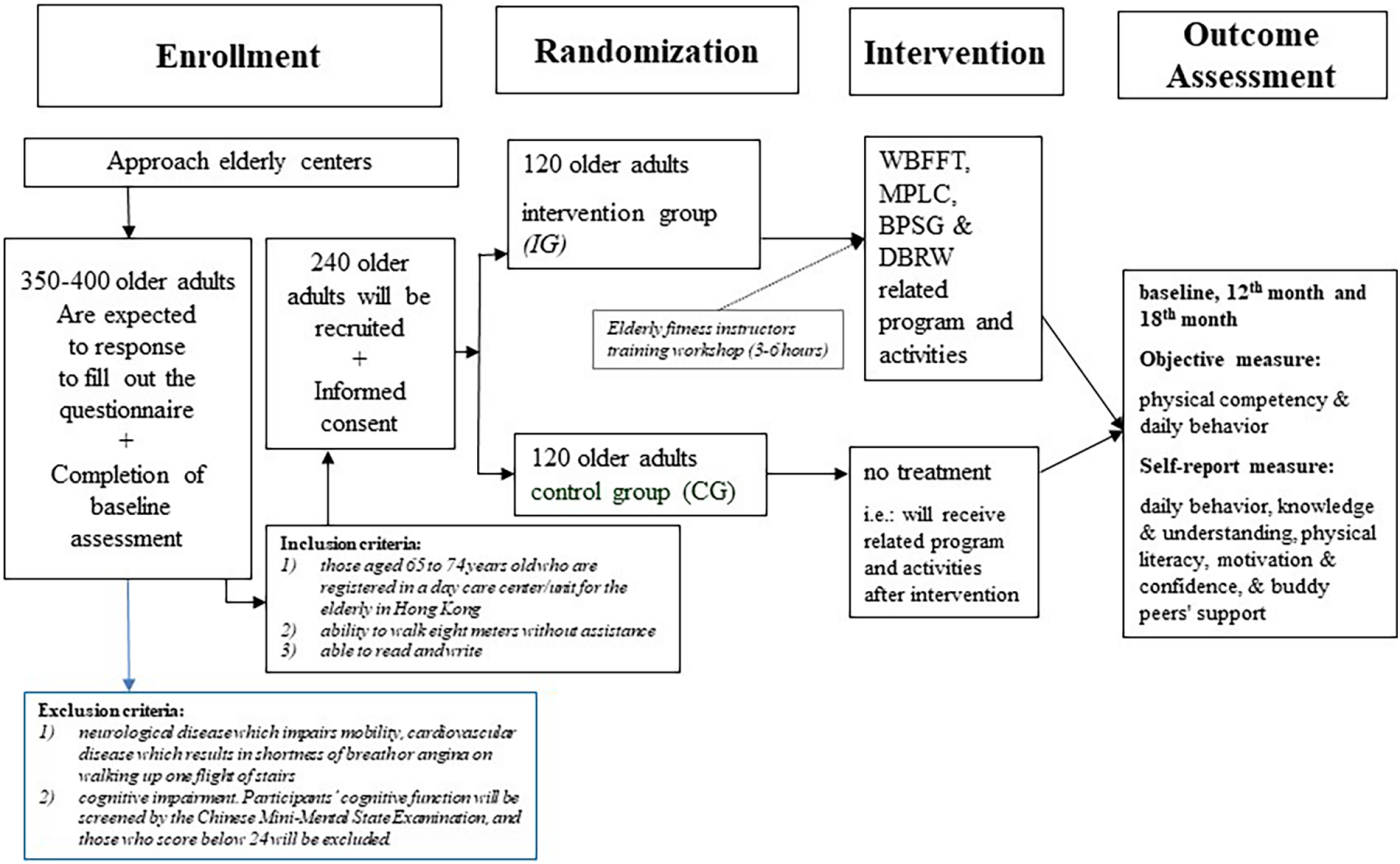
Flow diagram for the PLBI intervention using CONSORT guidelines for RCT.

A two-arm cluster randomized controlled trial will be employed in this proposed study (see details in Figure 1). According to the Social Welfare Department of Hong Kong, there are 98 daycare centers for the older adults in 2023 (11). Daycare centers for the older adults located in 3 regions in Hong Kong will be invited to participate in the intervention. The physical literacy-based intervention (PLBI) will be conducted for twelve weeks.

### Sample size calculation

The required sample size is calculated based on the hypothesized effect sizes, and the likely rates of participant drop-out for the outcomes. The estimated sample size is 198 with an effect size of 0.25, α of 0.05 and power of 0.95 (12). With the anticipation of a 20% participant drop-out rate, this led to a required number of 120 participants per group. Therefore, 10 daycare centers for the older adults in Hong Kong will be invited to participate in this study.

### Participants

Inclusion criteria will be: (1) those aged 65 to 74 years old who are registered in a daycare center/unit for the older adults in Hong Kong, (2) the ability to walk eight meters without assistance (13), and (3) able to read and write. Exclusion criteria will include (1) neurological disease which impairs mobility, cardiovascular disease which results in shortness of breath or angina on walking up one flight of stairs, and (2) cognitive impairment. Participants' cognitive function will be screened by the Chinese Mini-Mental State Examination (14, 15), and those who score below 24 will be excluded.

The research team will randomly send a series of email messages and invitation letters to daycare centers in accordance with their district via a computer-generated randomization sequence (GraphPad Software, Inc.) by a statistician who is blinded to the allocation of participating daycare centers. After the daycare centers agree to participate, a leaflet package, which describes the theoretical background, timeframe, and main objectives/learning goals, will be delivered to the staff of daycare centers. Then an introductory meeting with staff will be held to illustrate practical details related to the intervention. The proposed program will be publicized in the participating daycare centers through posters and pamphlets, where the efficacy and benefits of PLBI will be briefly outlined. Recruitment will follow by setting up counters supported by the staff in the participating centers. Those who express interest will have their eligibility screened through a short questionnaire based on the inclusion and exclusion criteria. More detailed information about the study will be given to the eligible participants. Written informed consent will be obtained.

### The descriptions of PLBI

The proposed study has adopted the draft Australian Sports Commission Physical Literacy Standard as a PLBI guideline within four domains (physical, psychological, social and cognitive), which could be applied throughout the course of an individual's life (16). Since little is known about the development of physical literacy among the older adults of Hong Kong and elsewhere in the world, the present study proposes to use a PLBI for developing physical literacy and to chart the physical literacy level among older adults in Hong Kong. In this connection, the following PLBI program and activities have been designed in the intervention of this proposed study.

#### Part 1: weekly-based functional fitness training

In this proposed study, participants will receive a 12-week duration of functional fitness training in daycare centers, which will be implemented twice a week (one during the week, another one at the weekend). Table 2 demonstrates a 12-week functional fitness training program specifically designed for older adults. The program incorporates the FITT principle (Frequency, Intensity, Time, and Type) to ensure an effective training experience. The exercises included in the program emphasize low-impact movements to minimize joint stress, and the progression is gradual to allow for adaptation and improvement over time. A further important issue that will be mentioned is related to ensuring the safety of older adults during the whole intervention, which will emphasize the process-oriented outcomes and encouragement to participation, rather than the product-oriented assessments. Meanwhile, the main goal of functional training is to improve the range of joint motion, increase muscle strength and flexibility, and boost blood circulation in a safe and acceptable manner. Therefore, symmetry training together with cardiovascular training is the core of the training program. Examples of the main training activities include cardio-full-body exercises (e.g., move adherence to the music training, including running, jogging, dancing movements, etc.), upper body exercises (e.g., bench press, rowing and lifting), lower body exercises (e.g., squatting and lateral hurdle jumps with tap-sensitive pods) and sensory integrative training (e.g., shooting training with an electronic sensory wall).

**Table 2:** 12-week functional fitness training program for older adults.

Week	Frequency (days/week)	Intensity (RPE^a^)	Time (minutes)	Type of exercise^b^ (low-impact exercise)	Progression
1	2	3–4	30–60	Stretching, seated exercises, gentle cardio training	Focus on learning proper form and technique, gradually increasing duration. Add one additional training item if deemed appropriate.
2			30–60	Stretching, seated exercises, gentle cardio training	
3			30–60	Stretching, gentle upper-body muscle and cardio training	
4		4–5	40–60	Revision of the previous session	Increase intensity slightly on the RPE scale. Increase exercise time by 10 min. Add one additional training item.
5			40–60	Same as the previous session, add brisk walking and dance movement	
6			40–60	Same as the above, jogging and dance movement	
7		5–6	50–60	Revision of the previous session	Increase intensity slightly on the RPE scale. Increase exercise time by 10 min. Add one additional training item.
8			50–60	Same as the previous session, add lower-body muscle training	
9			50–60	Same as the above	
10		6–7	60	Revision of the previous session	Increase intensity slightly on the RPE scale. Increase exercise time by 10 min. Maintain intensity and exercise time.
11			60	Same as the previous session, add sensory integrative training	
12			60	Same as the above	

^a^ RPE, rate of perceived exertion scale, where 1 represents very light intensity and 10 represents maximal exertion.

^b^ Type of Exercise: The training program will be modified according to participants’ adaptability and capability.

#### Part 2: mastering physical literacy class

A 30-min mastering physical literacy class program will be conducted by experienced older adults health and fitness instructors after the weekly-based functional fitness training. This program aims to enhance the knowledge and understanding of physical literacy. Referring to the newly developed consensus statement (17), the mastering physical literacy class includes the following five domains related to the relationship with movement and physical activity throughout life:

(i) Why physical literacy matters—improves health, well-being and quality of life.
(ii) Understanding physical literacy—value, enjoy and engage in physical activity for life.
(iii) Everyone's physical literacy is different—their individual needs and experiences of movement and physical activity.
(iv) Building physical literacy—think, feel, move and connect with others.
(v) How experience affects physical literacy—people, places and spaces around us.

#### Part 3: daily-based reflective writing

Evidence has shown that daily-based reflective writing method has the therapeutic benefits of emotional disclosure, not only among adults with chronic illness but also in healthy older adults (18). At the end of the baseline stage of this proposed study, each participant of the intervention group will receive a template notebook for daily-based reflective writing. The required columns consist of the daily diet, physical activity, mood and sleep quality. Participants will be required to write down their behavior in this notebook daily. In each week's functional training course, there will be a life-sharing session, where the participants will be reminded to bring their notebooks and share their daily activities with others. They will receive feedback, advice and encouragement from the instructor and each other. The notebook will be collected during the last week of the intervention.

#### Part 4: buddy peers support group

The buddy peers support group introduced in this program may greatly increase social support which will be shown to be a key determinant of adherence to the exercise programs and create a commitment to achieve specified levels of physical activity (19). Therefore in this proposed study, peer-support groups with buddy members will be arranged in each daycare center. Three pairs of buddies and hence about 6 participants will be formed per group. A total of 6 buddy pairs and 2 groups will be formed per daycare center. Buddy peers will encourage each other to do functional exercises regularly or to perform functional exercises together. The time and place for the practice will be decided among buddy members. A member in each group will be nominated as the leader to assist in the liaison and coordination of group activities. The group will perform weekly group-based functional exercises, and the time and place for the practice will be decided among group members. The group will be encouraged to hold monthly gatherings with their fitness instructor in order to strengthen social support.

### Control group

Participants will follow the inclusion and exclusion criteria of the PLBI group. Participants will not receive PLBI intervention treatment. However, participants in the control group will be given the same treatment (program and activities) after all data has been collected.

### Outcome measures

This study will adopt both objective and self-reported measures to cover the elements and domains of physical literacy. Participants will be evaluated at baseline (week 0), post-intervention (week 12), and at a 6-week follow-up (week 18). All of them will be conducted at daycare centers for the older adults. The self-reported measurements will be administered by research assistants while the objective measurements will be performed by certified physical fitness assessors and health professionals who are blinded to the randomization for avoiding bias.

#### Objective measure (primary outcomes)

##### Physical competence

The Short Physical Performance Battery (SPPB) will be used to measure the physical competence within the physical literacy of older adults (20). Huang et al. (15) suggested using a combined and comprehensive kit of assessment tools to measure physical competence, and the SPPB was reported to be able to measure complex capabilities with excellent test-retest reliability, especially in community-dwelling older adults. It is a group of measures that combines the results of the gait speed, chair stand and balance tests, which consist of balance, a timed eight-foot walk and chair stands. The scores range from zero (worst performance) to 12 (best performance). It has been used as a predictive tool for possible disability and can aid in monitoring function in older people. The reliability of SPPB has been reported as ICC = 0.75–0.89 for all measures.

##### Daily behavior

Accelerometers (Actigraph wGT3X-BT) will be used to measure the physical activity engagement levels of participants, and are categorized as sedentary, light, moderate and vigorous. Data will be collected in 60 s epochs to account for older adults' natural activity levels, as it has been shown to present the most acceptable classification accuracy for accelerometer use among older adults (21). The cut-points developed by Aguilar-Farías et al. will be applied to identifying intensity levels (21). Participants will wear accelerometers at the waist to measure their physical activity engagement levels for at least 8 h per day, for seven consecutive days.

#### Self-report measures (secondary outcomes)

##### Demographic information

Demographic information including age, gender, body mass index (BMI), height and weight, education, and socio-economic status will be included in the starting part of the questionnaire set, in order to acquire personal characteristics for further analysis.

##### Daily behavior

International Physical Activity Questionnaire—short form is the short version of the International Physical Activity Questionnaire used to measure self-reported physical activity levels. They will be required to report on the total duration of different types of physical activity which lasted at least 10 uninterrupted minutes in the last 7 days. Example item included: During the last 7 days, how many days did you do vigorous physical activities?

##### Knowledge and understanding

Montreal Cognitive Assessment (MoCA) is a 30-question brief and sensitive test used for detecting Alzheimer's disease and measuring executive functions and multiple cognitive domains. It is widely adopted for older adults and will be used to analyze its association with the knowledge and understanding of physical literacy among older adults. MoCA-B is a revised MoCA test. The Chinese Version of MoCA-B tests nine cognitive domains (executive function, language, orientation, calculation, conceptual thinking, memory, visual perception, attention, and concentration), and has been reported as a reliable cognitive screening test across all education levels in Chinese older adults (22), with high acceptance and good reliability.

##### Physical literacy

The perceived Physical Literacy Instrument (PPLI) is a 9-item instrument which is used to measure the perceived physical literacy of different individuals (23, 24). Three sub-scales are “sense of self and self-confidence”, “self-expression and communication with others” and “knowledge and understanding” which were identified as key attributes of physical literacy (4). Participants responded to the instrument on a 1 to 5 Likert scale (1 = strongly disagree and 5 = strongly agree). Sum et al. (23) confirmed that the three-factor validity (RMSEA = 0.08; CFI = 0.94 and SRMR = 0.04) and convergent validity (CR = 0.72–0.78; AVE = 0.43–0.54) of the PPLI was satisfactory.

##### Motivation and confidence

Perceived Well-being Scale (PWB) contains 14 items with 7 points which will be used to measure participants' motivation and confidence (25). It is a short and convenient measure for use with community-based and institutionalized older adults. Example items include: (a) I am often bored (psychological well-being); (b) I am in good shape physically (physical well-being). The validity of PWB is sufficiently high to justify being used with the older adults, in particular in longitudinal and intervention studies.

##### Buddy peers support

A 5-item Friend Support for Exercise Habit Subscale of the validated Social Support for Diet and Exercise Behavior Scale will be used (26). Five questions are “exercise with me”, “offered to exercise with me”, “gave me reminder to exercise”, “gave me encouragement to stick with my exercise program”, and “changed their schedule so we could exercise together”. Participants responded to the instrument on a 1 (none) to 5 (very often) Likert scale. Sallis et al. (26) confirmed that the sub-scales are with high acceptance and good reliability.

### Data analysis

SPSS version 28 for Windows will be used for data analysis. Internal consistency reliability coefficients (Cronbach alpha) will be calculated for all sub-scales within the psychometric questionnaires. A cut-off score of 0.7 will be used to determine acceptable reliability. Descriptive statistics (means and standard deviations) will be calculated for participants' primary and secondary outcomes (objective and self-reported) at three-time points. Multilevel modelling methods were used to account for the clustered nature of the data. Specifically, we used three-level (time within older adults within day care centre) regression analyses to examine the effects of group (experimental vs. control), time (baseline, post-test and follow-up), and the group–time interaction (i.e., “intervention effect”) on the primary and secondary outcomes. Covariates such as age, gender, BMI, education, and socio-economic status will be included in the analysis. Bonferroni *post hoc* analyses will be conducted to determine contributing factors to the significance of F values, and the confidence level will be set at 95% to maintain statistical reliability for all the analyses.

### Evaluation plan

At post-intervention, a group of 8–12 participants will be contacted to explore their experiences in participating in the intervention through the in-depth interviews, the level of satisfaction with the intervention, and the barriers and expediters in performing the PLBI as instructed. Daily-based reflective writing will also be material for evaluation analysis. The reasons why participants dropped out of the study will also be recorded. Additionally, participants will be invited to fill in a process evaluation survey which assesses their level of involvement and level of satisfaction with the intervention.

## Discussion

The proposed study will demonstrate the effectiveness of the development of physical literacy for older adults in Hong Kong. The project hypothesizes that higher levels of physical literacy are supposed to provide benefits both for individuals and social groups, and meantime it does not require large and complex equipment through the easy access of all the facilities. The above-mentioned PLBI mainly addresses several components within physical literacy through functional fitness training, mastering physical literacy class, buddy peer support, and reflective writing, while the target group was the focus of older adults. This aligns three key constructs being emerged with the need for purposeful activities, knowledge of age-related changes and social interactions identified in the previous integrative review of framing physical literacy for older adults (27).

In the current proposed study, we aim to explore specific physical literacy tasks (in regard to physical competency, daily behavior, knowledge and understanding, and motivation and confidence) among older adults in Hong Kong. Moreover, it is aimed to examine the effectiveness of the PLBI in terms of changes in physical literacy levels among older adults in Hong Kong. We believe such strategies could be tailored for this population by adding novel information to the literature on physical literacy, gerontology, and public health in the Asian context of Hong Kong. In the long run, the project has the potential to sustain and expand. With the informing policy on the care of older adults, there may be more functional fitness training centers designed and implemented in the community, which could relieve the government's heavy burden on social services provision and limited healthcare resources. Such strategies could work as compelling approaches to continue to contribute to the whole society's social cohesion.

While only one study adopting a non-cluster randomized controlled trial to evaluate holistic exercise training for ageing adults utilized physical literacy as the guiding framework when designing the intervention, their focus was not on older adults but rather on the inactive community-dwelling adults to improve their physical literacy (15). The current study provides a comprehensive design and exhaustive assessment protocol for improving their functional performances to resist ageing. Further, this would aim to assist self-monitoring, goal setting, and behavior change underpinning the concept of physical literacy among older adults. It is of great significance for future practical implementation that employing an appropriate approach to evaluate physical literacy for older adults (28), which is not only concerned with the holistic nature of the physical literacy journey but emphasizes its contribution towards healthy ageing that could link the concept of physical literacy and rehabilitation for older adults. As echoed with the recent integrative review (27), implementing rehabilitation exercises in adherence to the promotion of physical literacy, the thematic analysis has already presented evidence of identifying shared constructs that optimal function and mobility should be more valued than rehabilitation exercises themselves among older people.

This proposed protocol adds value to the limited literature on elaborating physical literacy as the conceptualized framework for implementing research among emerging adults and older adults (29), by highlighting the key components that are central to physical literacy for this vulnerable population. Future physical literacy interventions should be designed with a special focus on identifying the key components (i.e., acquiring motivation and confidence to the engagement in meaningful physical activities, obtaining knowledge of age-appropriate changes, and understanding adaptive changes coherently, etc.), and determining whether those components are important for optimal ageing. Most importantly, it is needed to disseminate the information and competent movement strategies through the promotion of the proposed project and make more influence for the future potential.

## Study limitations

As a novel intervention in an emerging area of research, the proposed PLBI design for employing a physical literacy project among older adults in Hong Kong is more theoretical than pragmatic due to limited literature to inform its real-world implementation. Although this study adopts a cluster randomized controlled trial design, the sample size calculation is based on the individual level rather than the cluster level. This could affect the power to detect differences between groups and limit the internal validity of the results. Despite the fact that in-depth interviews will be conducted with participants to evaluate the intervention, the current design does not adopt a mixed-method approach to data collection, this limits the depth of information that can be gathered to understand participants' experiences and the contextual factors that influence the intervention's effectiveness. Such interpretation of the results should be treated with caution. The study has limitations related to the novel intervention design, sample size calculations and reliance on quantitative data. Further refinement and pilot testing are needed to improve the intervention before a large-scale trial. Despite these limitations, the study aims to provide novel insights and inform the future development of physical literacy interventions for older adults.

## Conclusion

The proposed study is the first attempt at the development of a PLBI for older adults in Hong Kong. This study is of significance because physical literacy has a potentially powerful impact on older adults' sense of self and self-confidence, communication with others, knowledge and understanding of physical activity, and motivation and accomplishments, which are the core constructs underpinning physical literacy. The proposed study protocol will propose a theoretical model by which the policymakers in Hong Kong or even worldwide assist health care services for older people. In the meantime, the Social Welfare Bureau can assist in supporting the implementation of the physical literacy project and promote the dissemination for older adults. The proposed intervention design in this study may guide the development of future programs in developing physical literacy for older adults.
